# *YES1* and *MYC* Amplifications as Synergistic Resistance Mechanisms to Different Generation ALK Tyrosine Kinase Inhibitors in Advanced NSCLC: Brief Report of Clinical and Preclinical Proofs

**DOI:** 10.1016/j.jtocrr.2022.100278

**Published:** 2022-01-20

**Authors:** Roberta Minari, Samuel Valentini, Denise Madeddu, Andrea Cavazzoni, Silvia La Monica, Costanza Anna Maria Lagrasta, Roberto Bertorelli, Veronica De Sanctis, Paola Fassan, Cinzia Azzoni, Lorena Bottarelli, Caterina Frati, Letizia Gnetti, Francesco Facchinetti, Pier Giorgio Petronini, Roberta Alfieri, Alessandro Romanel, Marcello Tiseo

**Affiliations:** a Medical Oncology Unit, University Hospital of Parma, Parma, Italy; b Department of Cellular, Computational and Integrative Biology (CIBIO), University of Trento, Trento, Italy; c Department of Medicine and Surgery, University of Parma, Parma, Italy; d NGS Core Facility, Department of Cellular, Computational and Integrative Biology (CIBIO), University of Trento, Trento, Italy; e Unit of Pathological Anatomy, University Hospital of Parma, Parma, Italy; f Institut National de la Santé et de la Recherche Médicale (INSERM) U981, Gustave Roussy Cancer Campus, Université Paris Saclay, Villejuif, France

**Keywords:** NSCLC, ALK TKIs, Resistance mechanism, *YES1* amplification, *MYC* amplification

## Abstract

**Introduction:**

ALK tyrosine kinase inhibitors (TKIs) are the standard treatment for advanced ALK-positive NSCLC. Nevertheless, drug resistance inevitably occurs. Here, we report a case of a patient with metastatic ALK-positive lung adenocarcinoma with an impressive resistance to sequential treatment with ALK TKIs mediated by *YES1* and *MYC* amplification in a contest of epithelial-to-mesenchymal transition and high progressive chromosomal instability.

**Methods:**

The patient received, after chemotherapy and 7 months of crizotinib, brigatinib and lorlatinib with no clinical benefit to both treatments. A study of resistance mechanisms was performed with whole exome sequencing on different biological samples; primary cell lines were established from pleural effusion after lorlatinib progression.

**Results:**

At whole exome sequencing analysis, *YES1* and *MYC* amplifications were observed both in the pericardial biopsy and the pleural effusion samples collected at brigatinib and lorlatinib progression, respectively. Increasing chromosomal instability from diagnostic biopsy to pleural effusion was also observed. The addition of dasatinib to brigatinib or lorlatinib restored the sensitivity in primary cell lines; data were confirmed also in H3122_ALK-positive model overexpressing both *YES1* and *MYC*.

**Conclusions:**

In conclusion, *YES1* and *MYC* amplifications are candidates to justify a rapid acquired resistance to crizotinib entailing primary brigatinib and lorlatinib resistance. In this context, a combination strategy of ALK TKI with dasatinib could be effective to overcome a rapid resistance.

## Introduction

Patients with advanced NSCLC harboring ALK rearrangement experience extended benefit from sequential treatment with ALK tyrosine kinase inhibitors (TKIs), reaching survivals up to 5 years.^1^

Acquired resistance to ALK TKIs inevitably occurs and is often mediated by acquisition of secondary *ALK* mutations.^2^ Other resistance mechanisms mediated by activation of different bypass signaling pathways were described.^2^ Moreover, epithelial-to-mesenchymal transition (EMT)^2^ and small cell transformation have been reported.^2^

Intrinsic resistance implies the absence of ALK TKI activity, therefore leading to poor outcomes: its mechanisms are poorly understood and this represents an important gap in the field of ALK TKI resistance. Here, we present a case report of a young never-smoker woman affected by an ALK-positive NSCLC tumor who presented an impressive resistance to sequential treatment with ALK TKIs mediated by *YES1* and *MYC* amplifications in a complex context of EMT and high progressive chromosomal instability. Preclinical proofs of their role were provided both in primary cell lines and *in vitro* model.

## Materials and Methods

For the extensive protocol, see the Supplementary Materials.

## Results

### Case Report

**Figure 1:**
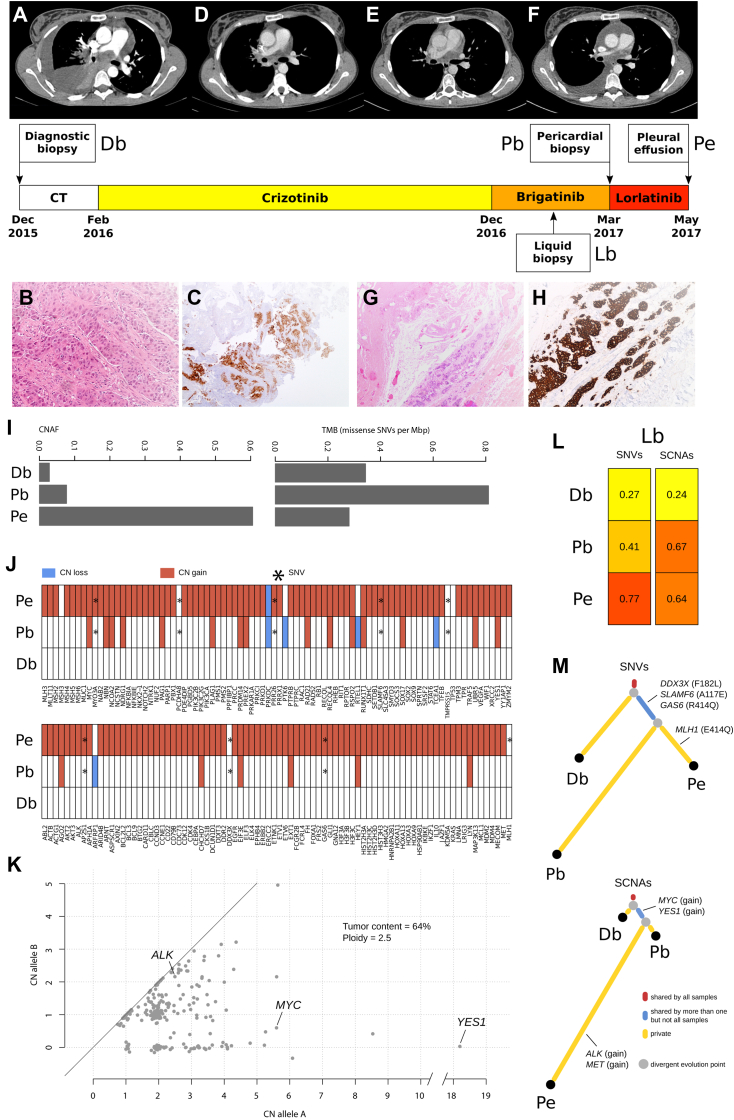
Patient’s clinical timeline with CT scan, histopathologic, and ALK examination results. (*A*) Baseline CT scan results with right pleural and pericardial effusion samples and multiple hilo-mediastinal bilateral lymph nodes. (*B*) H/E staining results of bronchial biopsy sample (20×) obtained at diagnosis and (*C*) result of ALK IHC (clone D5F3) positivity obtained with VENTANA DP 200 slide scanner (10×). (*D*) Tumor response after two courses of carboplatin and pemetrexed and (*E*) after crizotinib administration. (*F*) Progression of the disease after two months of brigatinib. (*G*) Result of H/E staining of pericardial biopsy sample and (*H*) confirmed ALK positivity with IHC (clone D5F3) obtained with VENTANA DP 200 slide scanner (10×). Molecular study of resistance. (*I*) Estimations of CNAF on the left and TMB on the right. TMB is calculated as the average of missense SNVs per captured Mbp, whereas CNAF is computed as the fraction of the genome with a log_2_ratio greater than 0.3 or less than (−0.3). (*J*) Landscape of genomic aberrations for a selection of genes, including common cancer drivers, recurrent CN aberrant genes in lung adenocarcinomas (cbioportal.org), MMR genes, and genes with missense SNVs shared by the pericardial biopsy and the pleural effusion. Each column represents a gene, and each row a tumor sample. Specifically, CN gains (log_2_ratio > 0.3) of ploidy-corrected genomic segments are represented in red, CN losses (log_2_ratio < −0.3) of ploidy-corrected genomic segments are represented in blue, whereas SNVs are represented with an asterisk. (*K*) Allele-specific CN analysis of the pleural effusion sample reveals distinct clusters of copy-neutral LOH (CN = 2|0), copy-aberrant LOH (CN = N|0 with N > 2), including *YES1* with an allele carrying 18 gene copies, and allele-specific gain (CN = N|M with N,M > 0 and N + M > 2), including *ALK* and *MYC*, with an allele carrying 5 copies. *YES1* and *MYC* amplifications were both confirmed by ddPCR analysis. The genomic profile is compatible with aneuploidy. (*L*) Fraction of SNVs and SCNAs detected in the cfDNA sample that are also detected, respectively, in the diagnostic biopsy, the pericardial biopsy, and the pleural effusion samples. (*M*) Phylogenetic trees built using SNVs (on the top) and SCNAs (on the bottom). Both trees reveal a branching evolution structure that follows the natural history of the disease. Somatic aberrations of interest are highlighted. Of note, a missense SNV private to the pleural effusion was identified in *MLH1* gene and missense SNVs in both the pericardial biopsy and the pleural effusion samples were found in *DDX3X*, *SLAMF6*, and *GAS6* genes. cfDNA, cell-free DNA; CN, copy number; CNAF, copy number aberrant fraction; CT, computed tomography; Db, diagnostic biopsy; ddPCR, digital droplet polymerase chain reaction; Dec, December; Feb, February; H/E, hematoxylin and eosin; IHC, immunohistochemistry; Lb, liquid biopsy; LOH, loss of heterozygosity; Mar, March; Mbp, mega base pair; MMR, mismatch repair; Pb, pericardial biopsy; Pe, pleural effusion; SCNA, somatic copy number alteration; SNV, single nucleotide variant; TMB, tumor mutational burden.

A 33-year-old never-smoker woman was hospitalized in December 2015 for dyspnea with findings of pleural and pericardial effusion owing to a right lung adenocarcinoma (Fig. 1*A* and *B*). Immunohistochemistry results revealed ALK positivity (Fig. 1*C*), confirmed with targeted RNA sequencing as EML4 (exon 13)–ALK (exon 20) variant 1. The patient received carboplatin-pemetrexed with tumor response after two courses (Fig. 1*D*); after the third cycle, dyspnea worsened and crizotinib was rapidly started in February 2016, with tumor response (Fig. 1*E*). After 7 months of crizotinib, subcarinal progression was treated with radiotherapy and crizotinib was continued. In December 2016, a pleuropericardial progression occurred with the need of pericardiocentesis which revealed ALK-positive adenocarcinoma cells. The second-generation ALK TKI brigatinib was administered from December 2016, without effect, as no clinical benefit was achieved and disease progression was documented after 2 months (Fig. 1*F*); a new pericardiocentesis was performed, followed by pleuro-pericardial window with a pleurocath positioning. The histologic results of pericardial localization confirmed ALK-positive adenocarcinoma (Fig. 1*G* and *H*). In March 2017, lorlatinib was started, but the the third-generation ALK TKI was ineffective with a rapid clinical worsening with a massive pleural effusion. Informed consent to perform molecular analysis in her pleural effusion sample and in other tissue biopsy samples was collected, but the patient passed away in May 2017.

### Molecular Study of Resistance

Tumor/normal whole exome sequencing (WES) was performed on all available tissue rebiopsy samples collected at progression of brigatinib and lorlatinib (pericardial biopsy and pleural effusion, respectively), on one liquid biopsy sample (collected during brigatinib treatment, 1 month before the pericardial biopsy), and on a matched control sample (Fig. 1). Single nucleotide variant analysis revealed low tumor mutational burden (<1 mutation per megabase) across all samples (Fig. 1*I*) and absence of secondary *ALK* mutations (Fig. 1*J*).

Somatic copy number alteration (SCNA) analysis across our samples revealed a temporally progressive chromosomal instability from the diagnostic biopsy to the pleural effusion samples (Fig. 1*I*). Gains for *YES1*, encoding a *SFK*, and *MYC* genes were observed both in the pericardial biopsy and the pleural effusion samples (Fig. 1*J*). An in-depth allele-specific copy number analysis of the pleural effusion sample revealed a complex genomic landscape (Fig. 1*K*) and highlights a strong copy-aberrant loss of heterozygosity of *YES1* gene and a *MYC* allele-specific gain.

Similarity analysis of single nucleotide variants and SCNA profiles between the liquid biopsy and the other samples (Fig. 1*L*), together with phylogenetic tree reconstructions (Fig. 1*M*), suggests a branching evolutionary tumor trajectory characterized by early dominant driver, emerging complex genomic landscape compatible with aneuploidy, and low background mutation rate.

**Figure 2:**
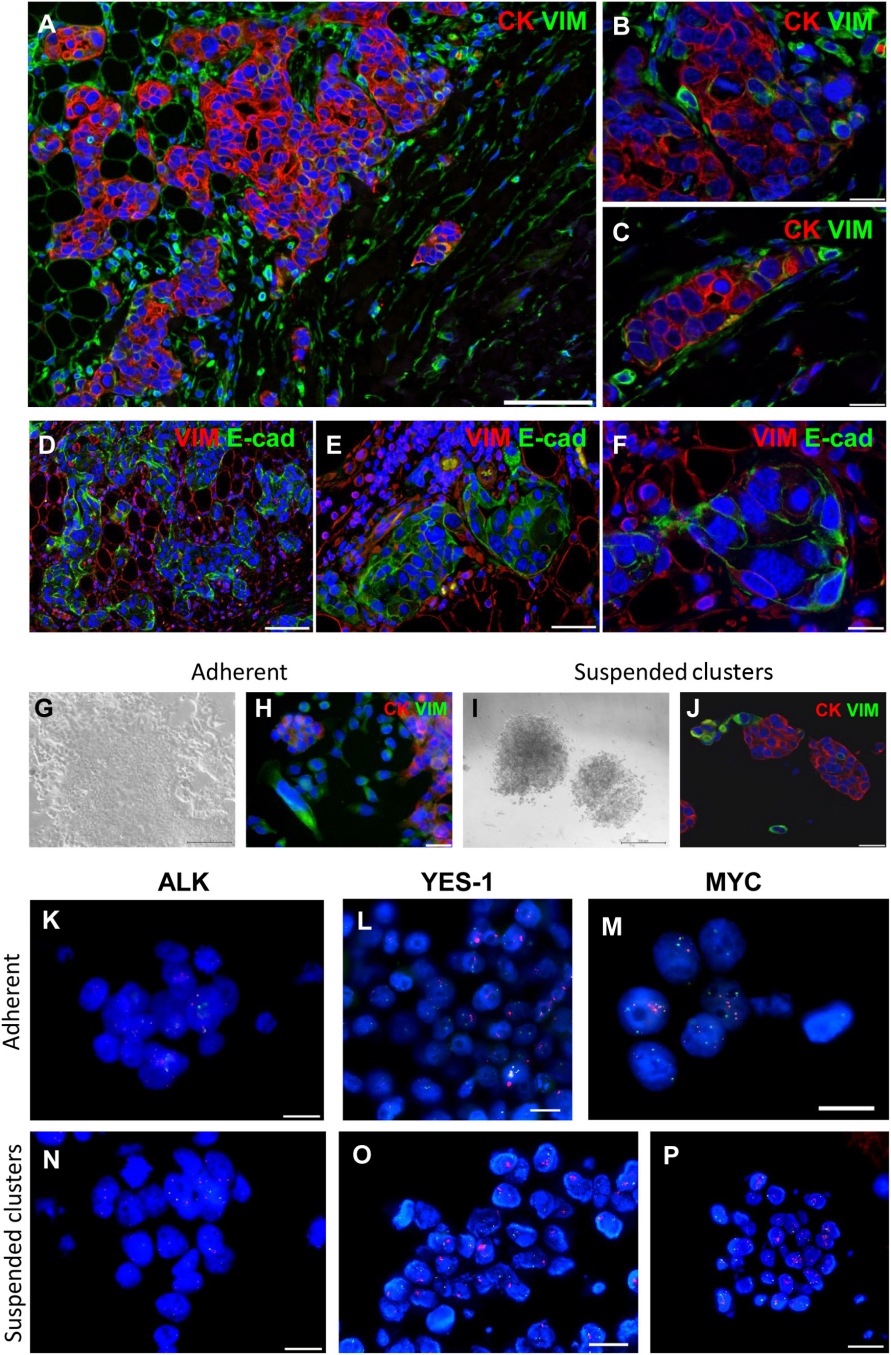
Immunohistochemical analysis of pericardial biopsy sample. (*A*–*C*) Section of immunostained pericardial infiltration of adenocarcinoma biopsy sample documenting, at different magnification, VIM-positive cells (green fluorescence) located at the edge of neoplastic glandular structure labeled by CK (red fluorescence). (*D*–*F*) Microphotographs, at different magnification, revealing the immunofluorescence characterization of pericardial biopsy sample in which E-cad (green fluorescence)–positive structures are surrounded by VIM-positive cells (red fluorescence) which confirmed the epithelial origin of the glandular structures in the pericardial tissue together with the presence of the peripheral cells displaying mesenchymal phenotype. Nuclei are revealed by the blue fluorescence of DAPI. Scale bars *A* and *D*: 100 μm; *B*, *C*, and *F*: 20 μm; and *E*: 50 μm. Characterization of the primary cell lines derived from pleural effusion sample. Phase-contrast images revealing (*G*) cultured adherent cells and (*I*) suspended clusters. Representative images of double immunostaining documenting CK (red fluorescence) expression in epithelial cells and VIM (green fluorescence)-labeled cells growing in (*H*) adherence or (*J*) located at the edge of CK-positive clusters. Nuclei are recognized by the blue fluorescence of DAPI. (*K* and *N*) ALK rearrangement, revealed by FISH analysis, is documented by the presence, in cell nuclei, of separated red and green dots. Representative images of FISH analysis revealing (*L* and *O*) *YES1* and (*M* and *P*) *MYC* amplification: gain of red dots (gene) over green dots (chromosome centromere) was presented. Blue fluorescence corresponds to DAPI counterstaining of the nuclei. Scale bars *A*: 200 μm; *B*: 500 μm; *C*: 20 μm; *D*: 30 μm; and *E*–*J*: 20 μm. CK, cytokeratin; DAPI, 4′,6-diamidino-2-phenylindole; E-cad, E-cadherin; FISH, fluorescence in situ hybridization; VIM, vimentin.

The pericardial biopsy sample was further characterized by immunohistochemical analysis to evaluate the expression of cytokeratin, E-cadherin, and vimentin (Vim), suggesting the gain of mesenchymal phenotype and the acquisition of migratory properties (Fig. 2*A*–*F*).

For the extensive protocol of the molecular study, see the Supplementary Materials.

### Preclinical Results

Primary cell lines were established from pleural effusion and double immunofluorescence staining samples for the epithelial markers EpCAM or cytokeratin, and the mesenchymal marker Vim was performed. The following two cell populations were distinguished: one adherent to the dish surface and the other spontaneously growing as spheroids (Fig. 2*G* and *I*). Adherent cells expressing Vim grew mainly as an independent population (Fig. 2*H*), whereas mesenchymal cells were mainly located at the periphery of cytokeratin-positive clusters or sprouted from spheroids corroborating the hypothesis of EMT (Fig. 2*J*). This observation prompted us to suppose that adherent mesenchymal cells could be already present among the epithelial cells in the pleural effusion sample and could be a result of EMT which occurred *in vivo*. In addition, retention of ALK rearrangement and presence of *YES1* and *MYC* amplification were evidenced by fluorescence in situ hybridization analysis in both populations (Fig. 2*K*–*P* and Supplementary Fig. 1*A* and *B*).

**Figure 3:**
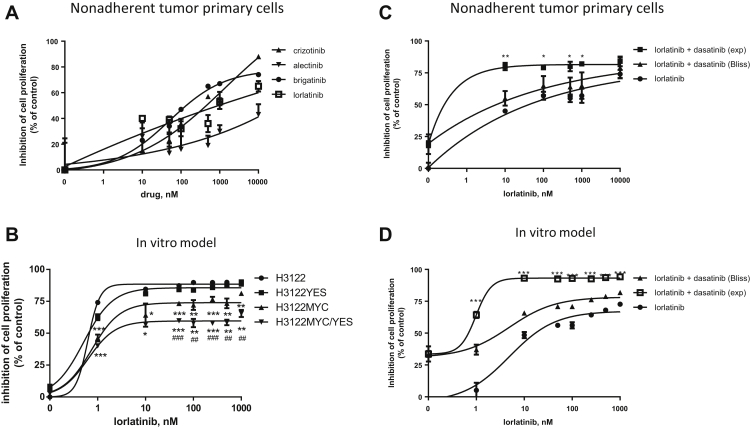
Effect of ALK inhibitors on cell proliferation in primary cell lines and in the *MYC/YES*–overexpressing cells. The nonadherent (*A*) primary cells were treated, with increasing concentrations of crizotinib, alectinib, brigatinib, and lorlatinib for 72 hours and then cell proliferation was evaluated by the MTS assay. Data are expressed as percentage inhibition of cell proliferation versus control cells and are means ± SD (n = 4). (*B*) H3122 parental (H3122), *MYC*–overexpressing (H3122MYC), *YES1*–overexpressing (H3122YES), and *MYC/YES1*–overexpressing (H3122MYC/YES) cells were treated with increasing concentrations of lorlatinib for 72 hours, and then cell proliferation was evaluated by crystal violet assay. Data are expressed as percentage inhibition of cell proliferation versus control cells and are means ± SDs; results are representative of three independent experiments (∗∗∗*p* < 0.001, ∗∗*p* < 0.01, ∗*p* < 0.05 versus H3122 and ^#^*p* < 0.05, ^##^*p* < 0.01, ^###^*p* < 0.001 versus H3122MYC). Src family kinase inhibition overcomes ALK inhibitor resistance in primary tumor cells and in the MYC/YES–overexpressing cells. The nonadherent primary cells were treated, with increasing concentrations of lorlatinib (*C*) in the absence or in the presence of 1 μM dasatinib. After 72 hours, cell proliferation was evaluated by MTS assay. Data are expressed as percentage inhibition of cell proliferation versus control cells and are means ± SD (n = 3) (∗∗∗∗*p* < 0.0001, ∗∗∗*p* < 0.001, ∗∗*p* < 0.01; Student’s *t* test). (*D*) The MYC/YES1–overexpressing cells were treated with increasing concentrations of lorlatinib in the absence or in the presence of 1 μM dasatinib. After 72 hours, cell proliferation was evaluated by crystal violet assay and the effect of the drug combinations was evaluated using the Bliss interaction model. Data are expressed as percentage inhibition of cell proliferation versus control cells and are means ± SDs; results are representative of three independent experiments (∗*p* < 0.05, ∗∗*p* < 0.01, ∗∗∗*p* < 0.001 versus Bliss theoretical). exp, experimental; MTS, 3-(4,5-Dimethylthiazol-2-yl)-5-(3-carboxymethoxyphenyl)-2-(4-sulfophenyl)-2H-tetrazolium.

We then evaluated the effect of ALK TKIs in inhibiting cell proliferation of primary cell lines. In respect to human EML4/ALK-rearranged H3122 cell line, sensitive to all ALK inhibitors (range concentration that inhibits 50%: 5–10 nM),^3^ our primary cell lines, either adherent (Supplementary Fig. 2*A*) or growing as spheroids (Fig. 3*A*), had resistance to these inhibitors.

To better define the role of *YES1* and *MYC* amplification in conferring resistance to ALK inhibitors, H3122 cells and *MYC*–overexpressing H3122 cells (H3122MYC)^4^ were infected with the lentiviral transfer vector carrying full-length human *YES1* cDNA (H3122MYC/YES) as previously described.^4^

Selected overexpressing clones were then analyzed for sensitivity to the alectinib, brigatinib, and lorlatinib. The sole overexpression of *YES1* did not modify the responsiveness to these inhibitors. By contrast, H3122MYC was more resistant to all the inhibitors, as already reported,^4^ and H3122MYC/YES had the highest resistance to the drugs (Fig. 3*B* and Supplementary Fig. 2*B* and *C*). With the aim to overcome ALK TKI resistance mediated by *MYC/YES1* amplification, we combined ALK inhibitors with dasatinib, tested either in the patient’s primary cell lines and in the H3122MYC/YES.

The combination of dasatinib with lorlatinib or with brigatinib restored the sensitivity to the ALK TKIs in primary cells, inducing an additive or synergistic effect in inhibiting cell proliferation (Fig. 3*C* and Supplementary Fig. 3*A*). Similarly, a strong synergistic effect was exerted when dasatinib was combined with ALK TKIs in H3122MYC/YES (Fig. 3*D* and Supplementary Fig. 3*B* and *C*), confirming the addiction of a SFK-targeting agent can overcome the resistance to ALK TKIs in the presence of the *MYC/YES1* amplification.

## Discussion

Here, we describe a case of a young never-smoker woman affected by advanced ALK-positive NSCLC who presented a dramatic clinical course to a sequential treatment with ALK TKIs, with an acquired resistance to crizotinib entailing primary resistance to brigatinib and lorlatinib. Results of WES analysis revealed the context of the disease with high genomic instability. The increasing level of SCNAs observed between the pericardial tissue and pleural effusion samples revealed a high aneuploidy profile that could lead to an aggressive tumor phenotype.

Analyzing more specifically the WES results, *YES1* and *MYC* amplifications may be implicated in the resistance to the ALK TKIs. *MYC* amplification was already described as a potential primary resistance mechanism to crizotinib in an ALK-positive patient with NSCLC.^4^ Regarding *YES1*, recently, Garmendia et al.^5^ presented a novel evidence for its amplification as a mediator of carcinogenesis, revealing its overexpression induced metastatic spread in *in vivo* models and that it is a predictive marker of dasatinib response in NSCLC cells. *YES1* is the only member of the SFK regulated mainly by gene amplification; it was described as a resistance mechanism to EGFR TKIs^6^ and recently reported in two of 17 ALK-positive patients with NSCLC as putative resistance mechanism to ALK TKIs.^6^

In our H3122MYC/YES model, we reported that *YES1* amplification seems not able alone to guide ALK TKI resistance; it needs a trigger, as *MYC* amplification, to generate high TKI-resistant cells. In contrast with our findings, Sato et al.^7^ reported that overexpression of either YES1 or YAP1 in ALK-positive cell lines conferred resistance to ALK TKIs. Nevertheless, according with that study, we observed that the addition of dasatinib to brigatinib or lorlatinib is able to restore sensitivity to ALK TKIs both in H3122MYC/YES and in primary cell lines.

Considering the absence of specific MYC inhibitor and the evidence that H3122MYC is highly sensitive to dasatinib,^8^ our results of restoration of ALK TKI sensitivity in the primary cell lines and in H3122MYC/YES could be explained by this broad activity of dasatinib both on YES1 and MYC.

*YES1* amplification is recently reported to be a noncanonical mechanism of YAP activation,^9^ which itself had a potential role in ALK TKI resistance.^10^^,^^11^ Moreover, this pathway was revealed to promote EMT in different cancer cell types.^12^^,^^13^ In our case, immunocytochemical characterization of pleural effusion revealed EMT features, confirmed also in primary cell line, highlighting the potential putative role of *YES1* in inducing EMT.

Furthermore, in our patient, mesenchymal microenvironment could have elicited genomic instability contributing to tumor evolution. Comaills et al.^14^ revealed that increased mesenchymal marker expression is correlated with genomic instability in circulating tumor cells of patients with metastatic breast cancer. On the basis of these evidences, *YES1* amplification could play a potential key role in inducing EMT and chromosomal instability and with the trigger played by *MYC* amplification in guiding resistance to ALK TKIs.

In conclusion, we provided an in-depth analysis of longitudinal samples derived from a patient with rapid resistance to three generations of ALK TKIs. *YES1* and *MYC* amplification, in a context of high genomic instability associated with EMT, are candidates to justify the rapid dramatic evolution. Prompt administration of ALK TKI in combination with dasatinib could be an effective strategy in the case of rapid ALK TKI resistance.

## CRediT Authorship Contribution Statement

**Roberta Minari**: Conceptualization, Writing - original draft, Writing - review & editing.

**Samuel Valentini**: Formal analysis, Methodology, Software.

**Denise Madeddu**, **Andrea Cavazzoni**, **Silvia La Monica**, **Costanza Anna Maria Lagrasta**: Formal analysis, Writing - original draft.

**Roberto Bertorelli**, **Veronica De Sanctis**: Formal analysis, Methodology.

**Paola Fassan**, **Cinzia Azzoni**, **LorenaBottarelli**, **Caterina Frati**, **Letizia Gnetti**: Formal analysis.

**Francesco Facchinetti**: Writing - original draft.

**Pier Giorgio Petronini**: Formal analysis, Methodology, Writing - original draft.

**Roberta Alfieri**: Conceptualization, Formal analysis, Writing - original draft.

**Alessandro Romanel**: Conceptualization, Formal analysis, Methodology, Software, Writing - original draft, Supervision.

**Marcello Tiseo**: Conceptualization, Writing - review & editing, Supervision.

## Acknowledgments

The authors thank the family of our young patient and “Verso il Sereno” association that supported our research. The H3122 cell line was kindly provided by Dr. Voena and Dr. Inghirami (Department of Molecular Biotechnology and Health Sciences, University of Torino, Torino, Italy). All authors have read and agreed to the published version of the manuscript.

## Informed Consent

Informed consent was obtained from the patient for publication of these case report and any accompanying images.
