# Supplementary material for: YES1 and MYC Amplifications as Synergistic Resistance Mechanisms to Different Generation ALK Tyrosine Kinase Inhibitors in Advanced NSCLC: Brief Report of Clinical and Preclinical Proofs
Source: JTO Clin Res Rep. 2022 Jan 20;3(2):100278. doi: 10.1016/j.jtocrr.2022.100278 (PMC8851257; doi:10.1016/j.jtocrr.2022.100278)
Supplement: Supplementary material [file mmc1.pdf]

Supplementary methods

Samples processing and DNA extraction and quantification

Tissue biopsies were microdissected in order to isolate normal from tumoral component and each sample was extracted with GeneRead DNA FFPE Kit (Qiagen, Venlo, Netherlands) following the manufacturer's instructions. Cell free DNA was extracted by plasma sample obtained by double centrifuge (2000g for 10') with QIAamp Circulating Nucleic Acid Kit (Qiagen).

Quantification and quality control were performed with Quantus (Promega, Madison, WI, USA) and TapeStation (Agilent, Wilmington, DE, USA) respectively.

Whole exome sequencing of tissue and cfDNA samples

Illumina compatible sequencing libraries were made following Takara ThruPLEX Tag-seq Kit and Nimblegen SEQCAP EZ Human Exome V3.0 library protocol for the enrichment, with some improvements [1]. In summary, from 100 to 150 ng of genomic DNA from tumoral tissue and matched control samples were sheared with the Diagenode NGS Bioruptor system whereas the cfDNA was only controlled for quality and quantity. All DNA fragments were purified and concentrated with Agencourt Ampure XP beads.

We prepared whole genome sequencing libraries from cfDNA from a cfDNA sample, tumoral samples and one matched control sample using ThruPLEX Tag-seq kit (Takara, Mountain View, CA, USA) as per manufacturer’s instructions. This kit introduces a 6 bp random molecular tag on both sides of DNA fragments to discriminate any non-reference alleles in the original template molecule from PCR duplication noise introduced during library preparation.

DNA was end-repaired and ThruPLEX stem-loop adapters with molecular tags were blunt-end ligated to the repaired input DNA. These molecules were extended and then amplified to yield molecularly tagged and sample-indexed Illumina NGS library.

We quantified the libraries and pooled them for target capture using Nimblegen SEQCAP EZ Human Exome V3.0 protocol except the use of IDT xGen Universal Blocking Oligos. Libraries were pooled at unequal concentration to obtain a high coverage of cfDNA and tumor sample libraries in comparison to control normal samples.

The amplified libraries were hybridized for 20 h with biotinylated oligo DNA baits for exome-containing libraries. The hybridized libraries were enriched with streptavidin-conjugated magnetic beads, washed and amplified by PCR (11 cycles), and the quality of the libraries was checked by qPCR as described in the protocol. The final libraries were sequenced on Illumina Hiseq2500 PE100 Rapid Run flowcells.

WES pre-processing and quality control

Raw FASTQ files obtained from cfDNA, three tumoral samples (corresponding to a diagnostic biopsy, a pericardial biopsy and a pleural effusion) and one matched control sample were processed for data analysis. FastQC tool was run on the raw reads to assess their quality [2]; quality metrics include average base quality, sequence duplication rate, and the k-mer enrichment along the length of the reads. These measures were utilized to assess whether the sequencing and the de-multiplexing of the samples was performed correctly.

After initial quality control and using an ad-hoc Perl script, UMI sequences of length 6bp were removed from raw paired-end reads and collected in a text file keeping the association to reads IDs. Raw reads stem sequences of length 11bp were removed resulting in final FASTQ files with reads of length 84bp. Reads were then aligned to GRC37/hg19 reference genome using BWA MEM [3].

Picard [4] and SAMtools [5] were used to generate single SAM files and an ad-hoc Perl script was implemented to re-introduce UMI sequences information in the created SAM files using an optional SAM format field BX of type Z. SAMtools were then used to generate sorted and indexed BAM files and Picard was used to remove duplicated reads exploiting UMI information by specifying READ_ONE_BARCODE_TAG=BX and READ_TWO_BARCODE_TAG=BX parameters. BAM files were then realigned (to correct for possible misalignments due to indels) and recalibrated (to adjust for over- and under-estimated base quality scores in the data) using GATK standard pipelines [6]. Finally, SAMtools were used to adjust BAM MD tags (strings for mismatch positions) and overlapping reads were clipped using the ClipOverlap module of BamUtil tool [7]. The alignment quality of the BAM files was obtained by several metrics related to the average coverage and capture rate to calculate how many aligned reads fall within a capture region of the Nimblegen SeqCap EZ Exome V3 kit. For any given sample, the capture rate was given by the percent of mapped reads that overlap any capture region in the kit and the total number of mapped reads. Average coverage was computed from the captured regions of the Nimblegen kit, resulting in an average depth of coverage in the captured regions of 137X for the cfDNA sample, 60X, 23X and 121X, respectively for the diagnostic biopsy, the pericardial biopsy and pleural effusion, and 42X for the matched control sample.

WES data segmentation

Segmentation of sample's sequencing data was performed using CNVKIT tool [8] and using the standard processing pipeline that exploits both on- and off-target reads to infer genomic segments and their log2(ratio). The same patient's control sample was used as reference to perform segmentation of all patient's cfDNA and tumoral tissue samples.

Detection of somatic single-nucleotide variants

To identify and characterize somatic single-nucleotide variants (SNVs) in WES captured regions, we applied a consensus approach combining three different SNVs detection tools: MuTect [9], Strelka2 [10] and SomaticSniper [11]. Putative somatic SNVs were nominated as genomic positions called by at least two out of the three tools and having a depth of coverage ≥10x, an allelic fraction (AF) ≥0.01, and a number of reads supporting the alternative base ≥2. Oncotator [12] was finally used to annotate retained SNVs with variant- and gene-centric information relevant to cancer. SNVs were considered of high-quality when AF was ≥0.05. Presence of secondary SNVs located in ALK coding regions was determined with a pileup approach using the PaCBAM tool [13]. No significant signal was identified.

Estimation of ploidy and tumor content

To assess tumor ploidy and tumor content from the tumoral samples and the cfDNA sample we used CLONET [14,15], a tool that combines copy number segmentation data and allelic-fraction imbalance at informative SNPs (i.e. germline heterozygous SNPs) to compute the estimations. Informative SNPs were determined using PaCBAM [13]. Ploidy was estimated for all samples, while tumor purity estimations were not available for the cfDNA sample, probably due to low ctDNA in circulation, and for the pericardial biopsy, due to low average depth of coverage.

Detection of somatic copy number aberrations

CLONET was used to compute ploidy-corrected somatic copy number aberrations (SCNAs) profiles, adjusting each segment to account for aneuploidy. Ploidy-corrected segments with mean log2(ratio) less than -0.3 or greater than 0.3 were categorized as copy-number loss or gain, respectively. For the pleural effusion sample, which showed aneuploidy (ploidy=2.5) and for which we also obtained an estimation of the tumor content (64%), we used CLONET to estimate allele-specific copy numbers. Specifically, CLONET transforms log2(ratio) values into cnB versus cnA values, where each segment is visualized at coordinates representing the number of copies of allele A (cnA) and allele B (cnB); in the absence of parental allelic information, cnA is considered ≥ cnB.

Phylogenetic analysis

Similarly to Faltas et al. [16], high-quality SNVs and ploidy-corrected SCNAs identified in the previous steps were used to reconstruct the phylogenetic tree of the patient using the parsimony ratchet method [17]. In this representation, each node models a population of tumor cells. Nodes with

no children, named leafs, represent cell populations from a tumor sampling, i.e., a tumor biopsy. Internal nodes model inferred tumor cell populations from observed SNVs and/or purified SCNAs. The node named WT represents a hypothetical population of wild-type cells (cells with no somatic aberrations). In phylogenetic trees, an edge connects two nodes; the length of an edge is proportional to the number of SNVs and/or purified SCNAs. A branch represents a time point in the evolution of the tumor where two distinct cell populations emerge; the length of the branches models the number of SNVs and/or purified SCNAs that are private to each population.

Similarity of tissues and cfDNA samples based on SNVs and SCNAs

For each pair (cfDNA, tumor sample) of patient's samples we computed the fraction of SNVs or genes with aberrant copy number (log2(ratio) value >0.3 or <-0.3) detected in the cfDNA sample that are also detected in the tumor sample.

RNA extraction, quantification and target RNA sequencing

RNA was extracted from tissue biopsies with MagCore HF16 Automated Nucleic Acid Extractor (RBC Bioscience) following the manufacturer's instructions.

RNA input quantification and degree of fragmentation of the samples were measured by real-time PCR on an EasyPGX qPCR instrument 96 (Diatech Pharmacogenetics, Jesi, Italy)

RNA libraries were generated using the Myriapod NGS Cancer panel RNA (Diatech Pharmacogenetics, Jesi, Italy), according to the manufacturers' instructions.

The RNA was retro-transcribed into cDNA using random hexamers. Subsequently, cDNA was amplified by multiplex-PCR using two primer mixtures to obtain fragments between 47 and 184 bases, including fusions of interest and endogenous control genes. The amplification products were purified with magnetic beads and barcoded by an amplification-based indexing reaction. The libraries thus achieved were normalized in quantity by magnetic beads to guarantee a homogeneous coverage of the samples during sequencing. Finally, the normalized libraries were mixed (library pool) and sequenced in parallel on the Illumina MiSeq platform (Illumina Inc., San Diego, CA, USA) with MiSeq Reagent Kit v2 Micro (300 cycles) flow cell (Illumina Inc., San Diego, CA, USA).

The data generated by the sequencer were analyzed locally with dedicated Myriapod NGS Data Analysis Software (v 4.0.2; Diatech Pharmacogenetics, Jesi, Italy).

Immunohistochemical analysis

Histologic sections of pericardial biopsy were exposed to double immunofluorescence labeling using anti-Cytokeratin (DAKO, Clones AE1/AE3, M3515, 1:50 o/n 4°C) and anti-Vimentin (Abcam,

ab92547, 1:100 90min 37°C). antibodies. Sections were then incubated with anti-mouse TRITC conjugated (1:20, 60min, 37°C, Jackson Laboratory, Baltimore, PA, USA) and anti-rabbit FITC conjugated (1:20, 60min, 37°C, Jackson Laboratory, Baltimore, PA, USA) secondary antibodies. Nuclei were counterstained with 4',6-diamidino-2-phenylindole (DAPI).

Cell Isolation

Pericardial fluid (90 ml) was collected aseptically and centrifuged at 1600 rpm for 5 min. After Red Blood Cells lysis, cell pellet was suspended in Dulbecco's Modified Eagle Medium (DMEM, Sigma Aldrich) added with 10% Fetal Bovine Serum (FBS, Sigma Aldrich), 1% Penicillin-Streptomycin (P/S, Sigma Aldrich) and 1% L-Glutamine (Sigma Aldrich) and seeded in 6 well plates at 37°C in a CO2 incubator (5% CO2/95% air). A fraction of the collected cells was fixed in 4% paraformaldehyde and employed for immuno-cytochemical analysis to verify the presence of cancer cells.

Within 10 days, two different cell populations could be distinguished: one growing as a monolayer adherent to the plate surface and another characterized by the presence of cell clusters growing in suspension. Cell populations were separated by collecting the supernatant, independently expanded and subsequently employed for the experiments.

Immuno-cytochemical analysis

EpCAM, Cytokeratin and Vimentin were detected in adherent cells and suspended clusters using immunofluorescence staining methods. Cells were fixed with 4% paraformaldehyde, cytospinned and exposed to primary antibodies.

Samples were incubated with primary antibodies against EpCAM (Abcam, ab32392, 1:50 o/n 4°C), Cytokeratin (DAKO, Clones AE1/AE3, M3515, 1:20 o/n 4°C) and Vimentin (Abcam, ab92547, 1:50 o/n 4°C). Specific FITC- or TRITC- conjugated (Sigma-Aldrich) secondary antibodies were employed. Nuclei were counterstained by DAPI.

Fluorescence In Situ Hybridization

Fluorescence In Situ Hybridization (FISH) was performed on both adherent cells and suspended clusters. Cells were fixed with 4% paraformaldehyde, cytospinned and pretreated in SSC 2x buffer for 30min at 80°C. ALK (ALK Break Apart FISH, ABBOTT VYSIS) rearrangements and *YES1* (YES1/CON18 FISH Probe Orange/Green, Empire Genomics) and *MYC* (MYC/Control 8 FISH Probe Orange/Green, Empire Genomics) amplifications were evaluated using specific probes and following manufacturer's instructions. ALK was considered rearranged when cell nuclei displayed

separated red and green dots. *YES1* and *MYC* were considered amplified when the ratio between Gene Copy Number (red dots) and Centromere (green dots) was ≥2.

Samples were viewed at 1000X magnification, employing a Nikon Ni-U fluorescence microscope (Nikon Corporation, Tokyo, Japan) equipped with an optic fiber. Images were acquired by the software NIS-Elements AR 5.01.00.

Cell culture and generation of YES-1 and MYC over-exressing cells by a lentiviral vector system

Human EML4-ALK rearranged NSCLC H3122 and *MYC*-overexpressing H3122 cells were cultured in Roswell Park Memorial Institute medium (RPMI 1640) supplemented with 2 mM glutamine, 10% fetal bovine serum (FBS), 100 U/mL penicillin, and 100 µg/mL streptomycin, as previously described [18]. The cells were incubated at 37°C in a humidified atmosphere containing 5% CO2.

Both parental and *MYC*-overexpressing H3122 cells were infected with the lentiviral transfer vector (pLenti-C-mGFP-P2A-Puro) carrying full length human *YES*-1 cDNA (Origene, Rockville, MD) as previously described [19] and clones selected by puromycin.

Drugs

Brigatinib, alectinib, crizotinib, lorlatinib and dasatinib were purchased from Selleck Biochemicals (Houston, TX, USA). The drugs were dissolved in DMSO, and DMSO concentration never exceeded 0.1% (v/v); equal amounts of the solvent were added to control cells.

Analysis of cell viability

Cells (5000/well) were plated in 100 µl per well in 96-well plates. Twenty-four hours after seeding, drugs were added in five replicates per concentration. After 72h, cell viability was measured with the CellTiter96®AQueous Non-radioactive Assay (Promega) in according to the manufacturer's instructions.

Western Blot Analysis

Procedures for protein extraction, solubilization, and protein analysis by 1-D PAGE are described elsewhere [20]. Western blotting was performed as previously described [21]. Antibodies against MYC, YES-1, and HRP-conjugated secondary antibodies were from Cell Signaling Technology Inc. (Beverly, MA, USA) and anti-actin (clone B11V08) was from BioVision (Milpitas, CA, USA). Primary antibodies were diluted 1:1000 in Tween-Tris Buffered Saline TTBS with 5% milk of BSA. Chemo-luminescence system (Immobilion™ Western Chemiluminescent HRP Substrate) was from Millipore. Reagents for electrophoresis and blotting analysis were from Bio-Rad. The chemiluminescent signal was acquired by the C-DiGit®Blot Scanner and the spots were quantified by the Image StudioTM Software (LI-COR Biotechnology, Lincoln, NE, USA).

Analysis of cell proliferation

Cell proliferation was evaluated by Crystal Violet (CV) staining as previously described [22]. The nature of the interaction between ALK inhibitors and dasatinib was calculated using the Bliss additivism model as previously described [23]. A theoretical dose-response curve was calculated for combined inhibition using the equation EBliss = EA + EB − EA*EB, where EA and EB are the percent of inhibition versus control obtained by ALK inhibitors (A) and dasatinib (B) alone and the EBliss is the percent of inhibition that would be expected if the combination was exactly additive. If the combination effect is higher than the expected EBliss value the interaction is synergistic, while if the effect is lower, the interaction is antagonistic. Otherwise, the effect is additive and there is no interaction between drugs.

Statistical Analysis

Statistical analyses were carried out using the GraphPad Prism 6.00 software. Comparisons were performed by the two-tailed Student's t-test, and p-values are indicated where appropriate.

References

1. Takara. Availabe online: http://takara.co.kr/file/manual/pdf/RDM-146-002-updated.pdf (accessed on 03/01/2021).
2. FastQC. Availabe online: www.bioinformatics.babraham.ac.uk/projects/fastqc. (accessed on 03/01/2021).
3. Li, H.; Durbin, R. Fast and accurate short read alignment with Burrows-Wheeler transform. *Bioinformatics (Oxford, England)* **2009**, *25*, 1754-1760, doi:10.1093/bioinformatics/btp324.
4. Picard. Availabe online: http://broadinstitute.github.io/picard/ (accessed on 03/01/2021).
5. Li, H.; Handsaker, B.; Wysoker, A.; Fennell, T.; Ruan, J.; Homer, N.; Marth, G.; Abecasis, G.; Durbin, R. The Sequence Alignment/Map format and SAMtools. *Bioinformatics (Oxford, England)* **2009**, *25*, 2078-2079, doi:10.1093/bioinformatics/btp352.
6. DePristo, M.A.; Banks, E.; Poplin, R.; Garimella, K.V.; Maguire, J.R.; Hartl, C.; Philippakis, A.A.; del Angel, G.; Rivas, M.A.; Hanna, M., et al. A framework for variation discovery and genotyping using next-generation DNA sequencing data. *Nature genetics* **2011**, *43*, 491-498, doi:10.1038/ng.806.
7. Jun, G.; Wing, M.K.; Abecasis, G.R.; Kang, H.M. An efficient and scalable analysis framework for variant extraction and refinement from population-scale DNA sequence data. *Genome research* **2015**, *25*, 918-925, doi:10.1101/gr.176552.114.

8. Talevich, E.; Shain, A.H.; Botton, T.; Bastian, B.C. CNVkit: Genome-Wide Copy Number Detection and Visualization from Targeted DNA Sequencing. *PLoS computational biology* **2016**, *12*, e1004873, doi:10.1371/journal.pcbi.1004873.
9. Cibulskis, K.; Lawrence, M.S.; Carter, S.L.; Sivachenko, A.; Jaffe, D.; Sougnez, C.; Gabriel, S.; Meyerson, M.; Lander, E.S.; Getz, G. Sensitive detection of somatic point mutations in impure and heterogeneous cancer samples. *Nature biotechnology* **2013**, *31*, 213-219, doi:10.1038/nbt.2514.
10. Kim, S.; Scheffler, K.; Halpern, A.L.; Bekritsky, M.A.; Noh, E.; Källberg, M.; Chen, X.; Kim, Y.; Beyter, D.; Krusche, P., et al. Strelka2: fast and accurate calling of germline and somatic variants. *Nature methods* **2018**, *15*, 591-594, doi:10.1038/s41592-018-0051-x.
11. Larson, D.E.; Harris, C.C.; Chen, K.; Koboldt, D.C.; Abbott, T.E.; Dooling, D.J.; Ley, T.J.; Mardis, E.R.; Wilson, R.K.; Ding, L. SomaticSniper: identification of somatic point mutations in whole genome sequencing data. *Bioinformatics (Oxford, England)* **2012**, *28*, 311-317, doi:10.1093/bioinformatics/btr665.
12. Ramos, A.H.; Lichtenstein, L.; Gupta, M.; Lawrence, M.S.; Pugh, T.J.; Saksena, G.; Meyerson, M.; Getz, G. Oncotator: cancer variant annotation tool. *Human mutation* **2015**, *36*, E2423-2429, doi:10.1002/humu.22771.
13. Valentini, S.; Fedrizzi, T.; Demichelis, F.; Romanel, A. PaCBAM: fast and scalable processing of whole exome and targeted sequencing data. *BMC genomics* **2019**, *20*, 1018, doi:10.1186/s12864-019-6386-6.
14. Prandi, D.; Baca, S.C.; Romanel, A.; Barbieri, C.E.; Mosquera, J.M.; Fontugne, J.; Beltran, H.; Sboner, A.; Garraway, L.A.; Rubin, M.A., et al. Unraveling the clonal hierarchy of somatic genomic aberrations. *Genome biology* **2014**, *15*, 439, doi:10.1186/s13059-014-0439-6.
15. Romanel, A.; Gasi Tandefelt, D.; Conteduca, V.; Jayaram, A.; Casiraghi, N.; Wetterskog, D.; Salvi, S.; Amadori, D.; Zafeiriou, Z.; Rescigno, P., et al. Plasma AR and abiraterone-resistant prostate cancer. *Science translational medicine* **2015**, *7*, 312re310, doi:10.1126/scitranslmed.aac9511.
16. Faltas, B.M.; Prandi, D.; Tagawa, S.T.; Molina, A.M.; Nanus, D.M.; Sternberg, C.; Rosenberg, J.; Mosquera, J.M.; Robinson, B.; Elemento, O., et al. Clonal evolution of chemotherapy-resistant urothelial carcinoma. *Nature genetics* **2016**, *48*, 1490-1499, doi:10.1038/ng.3692.
17. Nixon, K.C. The Parsimony Ratchet, a New Method for Rapid Parsimony Analysis. *Cladistics* **1999**, *15*, 407-414, doi:https://doi.org/10.1006/clad.1999.0121.

18. Rihawi, K.; Alfieri, R.; Fiorentino, M.; Fontana, F.; Capizzi, E.; Cavazzoni, A.; Terracciano, M.; La Monica, S.; Ferrarini, A.; Buson, G., et al. MYC Amplification as a Potential Mechanism of Primary Resistance to Crizotinib in ALK-Rearranged Non-Small Cell Lung Cancer: A Brief Report. *Translational oncology* **2019**, *12*, 116-121, doi:10.1016/j.tranon.2018.09.013.
19. Fumarola, C.; Cretella, D.; La Monica, S.; Bonelli, M.A.; Alfieri, R.; Caffarra, C.; Quaini, F.; Madeddu, D.; Falco, A.; Cavazzoni, A., et al. Enhancement of the anti-tumor activity of FGFR1 inhibition in squamous cell lung cancer by targeting downstream signaling involved in glucose metabolism. *Oncotarget* **2017**, *8*, 91841-91859, doi:10.18632/oncotarget.19279.
20. Cavazzoni, A.; Alfieri, R.R.; Carmi, C.; Zuliani, V.; Galetti, M.; Fumarola, C.; Frazzi, R.; Bonelli, M.; Bordi, F.; Lodola, A., et al. Dual mechanisms of action of the 5-benzylidene-hydantoin UPR1024 on lung cancer cell lines. *Molecular cancer therapeutics* **2008**, *7*, 361-370, doi:10.1158/1535-7163.mct-07-0477.
21. Cavazzoni, A.; Alfieri, R.R.; Cretella, D.; Saccani, F.; Ampollini, L.; Galetti, M.; Quaini, F.; Graiani, G.; Madeddu, D.; Mozzoni, P., et al. Combined use of anti-ErbB monoclonal antibodies and erlotinib enhances antibody-dependent cellular cytotoxicity of wild-type erlotinib-sensitive NSCLC cell lines. *Molecular cancer* **2012**, *11*, 91, doi:10.1186/1476-4598-11-91.
22. Alfieri, R.R.; Galetti, M.; Tramonti, S.; Andreoli, R.; Mozzoni, P.; Cavazzoni, A.; Bonelli, M.; Fumarola, C.; La Monica, S.; Galvani, E., et al. Metabolism of the EGFR tyrosin kinase inhibitor gefitinib by cytochrome P450 1A1 enzyme in EGFR-wild type non small cell lung cancer cell lines. *Molecular cancer* **2011**, *10*, 143, doi:10.1186/1476-4598-10-143.
23. La Monica, S.; Galetti, M.; Alfieri, R.R.; Cavazzoni, A.; Ardizzoni, A.; Tiseo, M.; Capelletti, M.; Goldoni, M.; Tagliaferri, S.; Mutti, A., et al. Everolimus restores gefitinib sensitivity in resistant non-small cell lung cancer cell lines. *Biochemical pharmacology* **2009**, *78*, 460-468, doi:10.1016/j.bcp.2009.04.033.
