# Supplementary material for: YES1 and MYC Amplifications as Synergistic Resistance Mechanisms to Different Generation ALK Tyrosine Kinase Inhibitors in Advanced NSCLC: Brief Report of Clinical and Preclinical Proofs
Source: JTO Clin Res Rep. 2022 Jan 20;3(2):100278. doi: 10.1016/j.jtocrr.2022.100278 (PMC8851257; doi:10.1016/j.jtocrr.2022.100278)
Supplement: Supplemental Figures [file mmc2.pdf]

# Supplementary Figure 1

A

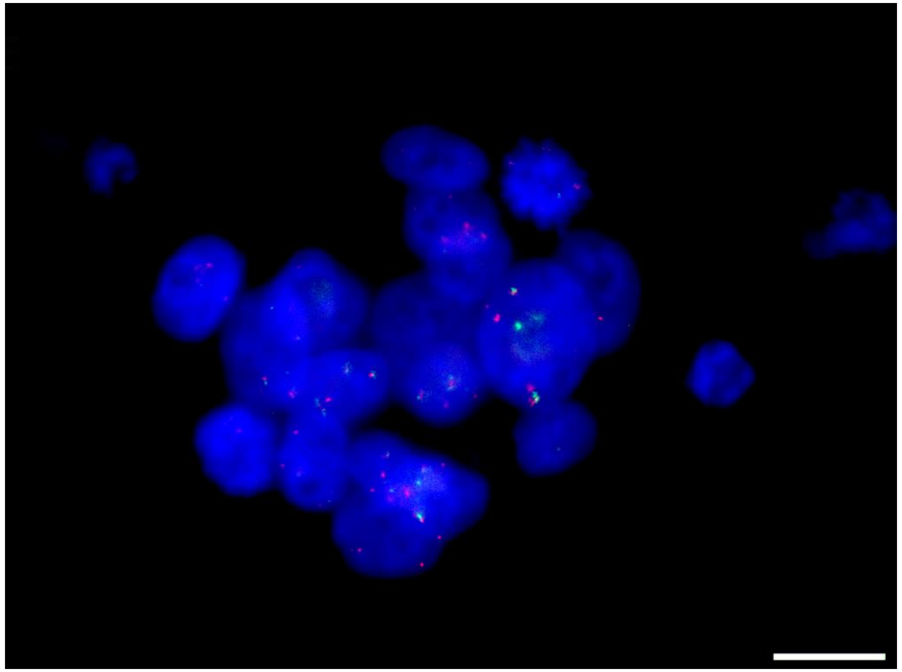

**A)** Enlarged representative images of Fluorescence In Situ Hybridization (FISH) analysis where ALK rearrangement is documented by the presence, in cell nuclei of adherent primary cell lines, of separated red and green dots.

B

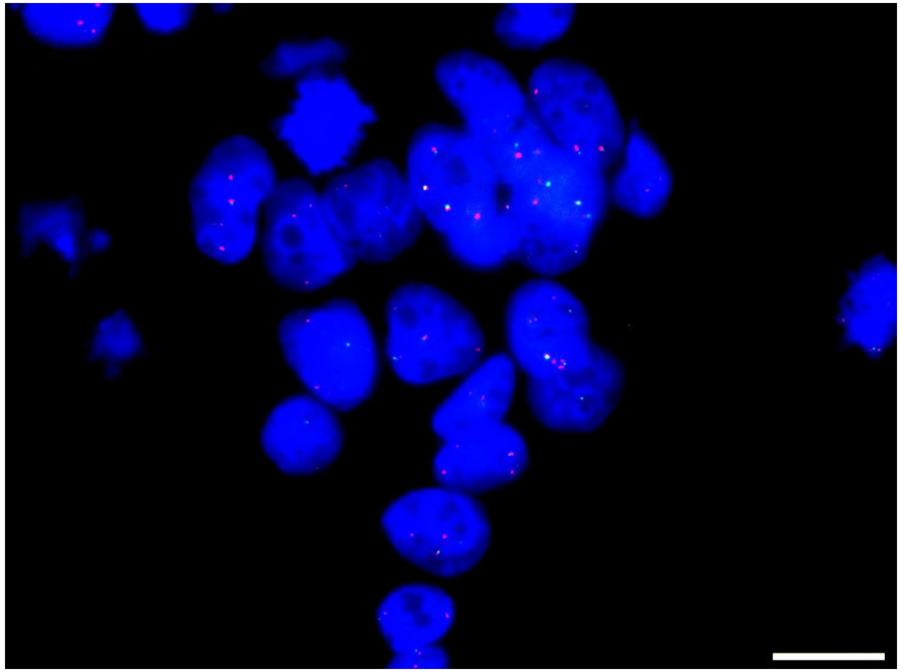

**B)** Enlarged representative images of Fluorescence In Situ Hybridization (FISH) analysis where ALK rearrangement is documented by the presence, in cell nuclei of suspended cluster of primary cell lines, of separated red and green dots.

# Supplementary Figure 2

**A**

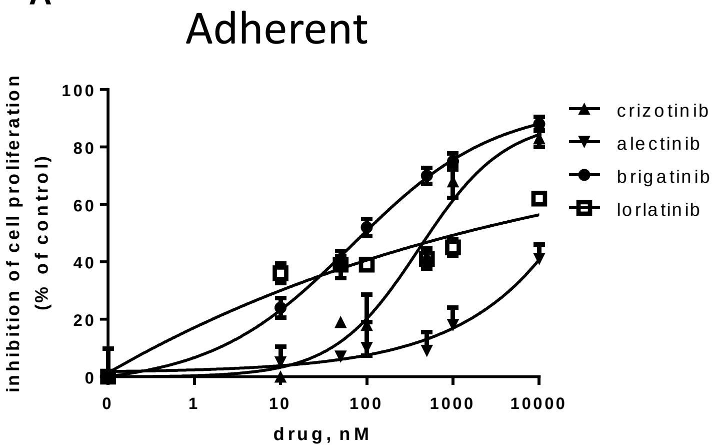


The adherent **(A)** primary cells were treated, with increasing concentrations of crizotinib, alectinib, brigatinib and lorlatinib for 72h and then cell proliferation was assessed by MTS assay. Data are expressed as percent inhibition of cell proliferation vs. control cells and are means ± standard deviation (SD) (N=4)

**B**

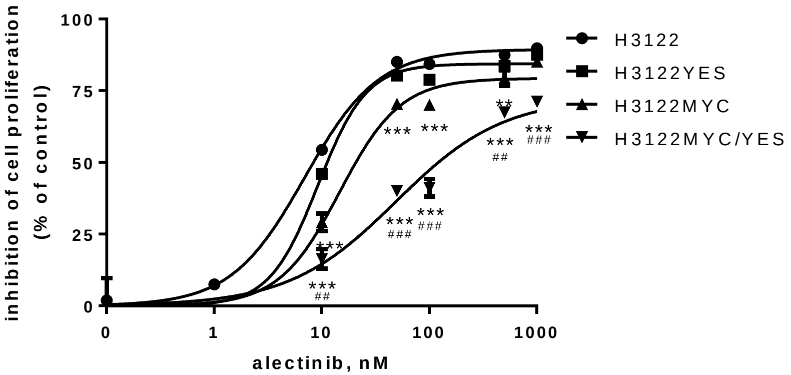


**C**

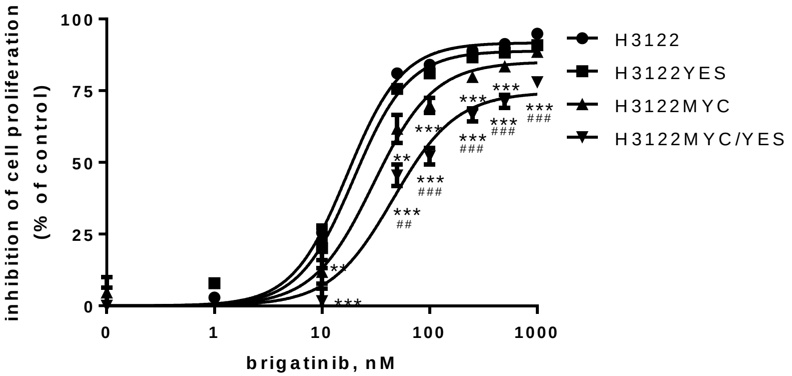


H3122 parental, *MYC*-overexpressing (H3122MYC), *YES1*-overexpressing (H3122YES) and *MYC/YES1*-overexpressing (H3122MYC/YES) cells were treated with increasing concentrations of alectinib **(B)** and brigatinib **(C)** for 72h, then cell proliferation was assessed by crystal violet assay. Data are expressed as percent inhibition of cell proliferation vs. control cells and are means ± SDs; results are representative of three independent experiments (***p<0.001, **p<0.01, *p<0.05 vs H3122; #p<0.05, ##p<0.01, ###p<0.001 vs H3122MYC).

Supplementary Figure 3

A

Non-Adherent

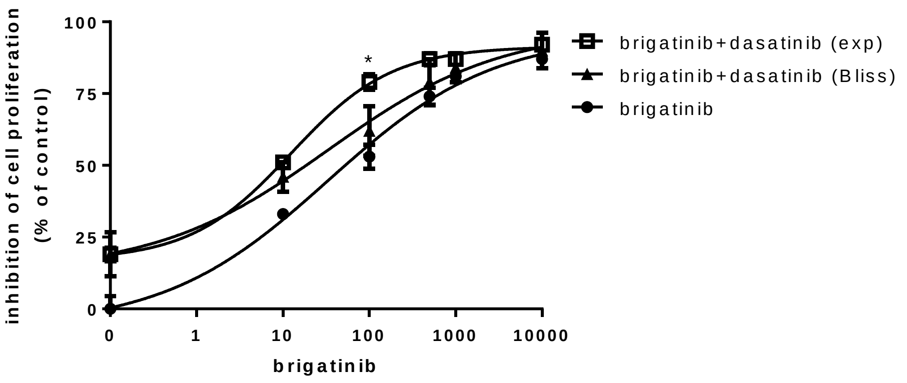


The non-adherent primary cells were treated, with increasing concentrations of brigatinib **(A)** in the absence or in the presence of 1µM dasatinib. After 72h cell proliferation was assessed by MTS assay. Data are expressed as percent inhibition of cell proliferation vs. control cells and are means ± standard deviation (SD) (N=3). (****p < 0.0001, ***p < 0.001, **p < 0.01; Student's t test).

B

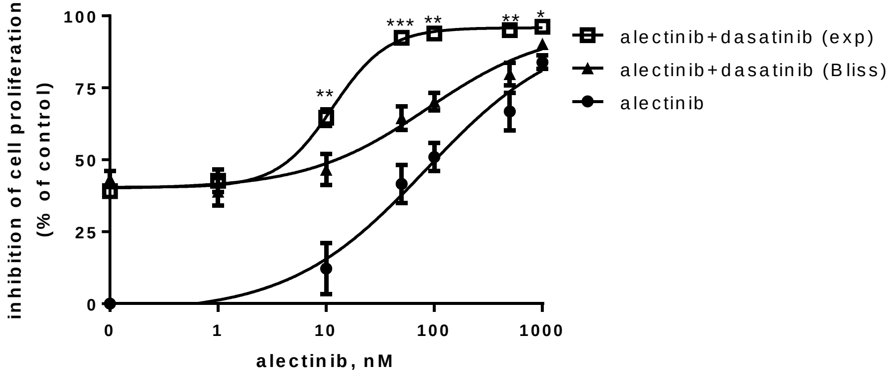


C

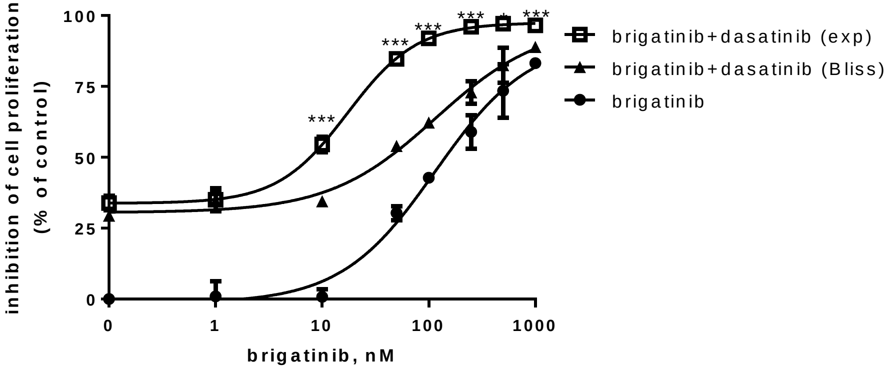


The *MYC/YES1*-overexpressing cells were treated with increasing concentrations of alectinib (B) and brigatinib (C) in the absence or in the presence of 1µM dasatinib. After 72h cell proliferation was assessed by crystal violet assay and the effect of the drug combinations was evaluated using the Bliss interaction model. Data are expressed as percent inhibition of cell proliferation vs. control cells and are means ± SDs; results are representative of three independent experiments (*p<0.05; **p<0.01; ***p<0.001 vs Bliss theoretical).
